# BCL2 regulates antibacterial autophagy in the intestinal epithelium

**DOI:** 10.1073/pnas.2410205121

**Published:** 2024-11-27

**Authors:** Yun Li, Shai Bel, Jamaal L. Benjamin, Kelly A. Ruhn, Brian Hassell, Cassie L. Behrendt, Zheng Kuang, Lora V. Hooper

**Affiliations:** ^a^Department of Immunology, University of Texas Southwestern Medical Center, Dallas, TX 75390; ^b^HHMI, University of Texas Southwestern Medical Center, Dallas, TX 75390

**Keywords:** autophagy, innate immunity, pathogenic bacteria, intestinal epithelium, enterocyte

## Abstract

Autophagy is a key innate immune defense mechanism in intestinal epithelial cells. Bacterial invasion of epithelial cells activates antibacterial autophagy through a process that requires the innate immune adaptor protein MYD88, yet how MYD88 signaling connects to the autophagy machinery is unknown. Here, we show that the mouse intestinal pathogen *Salmonella* enterica Serovar Typhimurium (*Salmonella* Typhimurium) triggers MYD88 signaling that regulates binding of the anti-autophagy factor B cell lymphoma 2 (BCL2) to the essential autophagy protein Beclin1 (BECN1) in small intestinal enterocytes, a key epithelial cell lineage. *Salmonella* infection activated the kinase c-Jun N-terminal protein kinase 1 (JNK1) downstream of MYD88. JNK1 induced enterocyte BCL2 phosphorylation, promoting dissociation of the inhibitory BCL2–BECN1 complex and releasing BECN1 to initiate autophagy. Mice with BCL2 phosphorylation site mutations that prevent BCL2–BECN1 dissociation showed increased *Salmonella* invasion of enterocytes and dissemination to extraintestinal sites. These findings reveal that BCL2 links MYD88 signaling to enterocyte autophagy initiation, providing mechanistic insight into how invading bacteria trigger autophagy in the intestinal epithelium.

## Significance

The epithelial cells that line the intestinal surface frequently defend against invading pathogenic bacteria. For example, the food-borne pathogen *Salmonella* enters the gut epithelial cell cytoplasm as a first step in causing disease. Autophagy is mechanism of antibacterial defense in which cytoplasmic bacteria are engulfed by membranes and routed to the lysosome for degradation. In this study, we identify a cellular pathway by which *Salmonella* triggers autophagy in mouse intestinal epithelial cells. The anti-autophagy protein B cell lymphoma 2 (BCL2) is central to this pathway by receiving input from innate immune bacterial detection pathways and relaying this input to the autophagy machinery. Our findings provide molecular insight into how bacteria induce autophagy in the epithelial lining of the intestine.

The intestinal epithelium interfaces with the diverse community of bacteria in the gut. To limit bacterial attachment and invasion, intestinal epithelial cells deploy an array of innate immune defense mechanisms. One of these mechanisms is antibacterial autophagy, in which cytosolic bacteria are marked and targeted to lysosomes for degradation (1–4). In mice, invasive intestinal bacteria activate gut epithelial cell autophagy which restricts intracellular bacteria and reduces their extraintestinal spread (5, 6). However, the mechanisms by which bacteria activate intestinal epithelial cell autophagy remain poorly understood.

The general process of autophagy involves engulfment of cytoplasmic materials—misfolded proteins, damaged organelles, or intracellular microorganisms—by double-membrane structures called autophagosomes (7, 8). Autophagosomes then fuse with lysosomes to deliver their contents for degradation (7, 8). The autophagy pathway involves the coordinated action of several proteins including the essential autophagy protein Beclin1 (BECN1). BECN1 forms a multimeric complex with vacuolar protein sorting 34 (Vps34) and class 3 phosphatidylinositol 3-kinase (PI3K) to nucleate autophagic vesicles and initiate autophagy (9, 10).

Autophagy occurs at a basal level in all cells but can be further induced by metabolic stressors such as starvation, allowing recovery of essential nutrients (11). Starvation initiates autophagy through well-characterized molecular mechanisms. In the absence of a metabolic stimulus, the antiapoptotic protein B cell lymphoma 2 (BCL2) complexes with BECN1 to inhibit autophagy (12). Nutritional stress induced by starvation (12) or exercise (13) triggers dissociation of the inhibitory BCL2–BECN1 complex, thus liberating BECN1 to initiate autophagosome formation through Vps34 and PI3K (12, 14). This mechanism involves phosphorylation of BCL2 at three residues by c-Jun N-terminal protein kinase 1 (JNK1) (15), which induces BCL2 dissociation from BECN1 and promotes autophagosome nucleation. Thus, BCL2 phosphorylation is essential for autophagy induction by metabolic stimuli.

We and others previously found that the invasive intestinal pathogen *Salmonella enterica* serovar Typhimurium (*S.* Typhimurium induces autophagy in mouse small intestinal epithelial cells, which restricts bacterial dissemination to other tissues (5, 6). Autophagy activation is a rapid and transient response to oral *S.* Typhimurium infection that peaks at ~24 h postinfection and diminishes rapidly thereafter (5). Invasion of *S.* Typhimurium into mouse epithelial cells is necessary to initiate autophagy (5), consistent with studies in cultured cells (3). Although large numbers of commensal bacteria also reside in the intestine, most resident bacterial species are not sufficiently invasive to activate robust autophagy in a wild-type host (5). Importantly, induction of epithelial cell autophagy by *S.* Typhimurium requires epithelial cell-intrinsic signaling through MYD88, an adaptor protein for multiple Toll-like receptors (TLRs) (5). Studies of *S.* Typhimurium infection of cultured cells have defined how intracellular bacteria are “marked” for autophagy (2, 3). However, it remains unclear how MYD88 signaling integrates with the autophagy machinery to initiate antibacterial autophagy in the mammalian intestinal epithelium.

Here, we show that BCL2 links MYD88 signaling to the epithelial cell autophagy pathway. We find that *S.* Typhimurium infection of the intestine induces phosphorylation of BCL2, promoting dissociation of the inhibitory BCL2–BECN1 complex and releasing BECN1 to initiate autophagy. We further show that epithelial cell BCL2 is phosphorylated by the kinase JNK1, which is activated downstream of MYD88, and that MYD88 thus regulates dissociation of the BCL2–BECN1 complex. Finally, we show that BCL2 phosphorylation is essential to limit intracellular bacteria in mouse enterocytes. Our findings reveal that BCL2 connects MYD88 signaling with the epithelial cell autophagy pathway and show that metabolic and microbial stimuli activate autophagy through common mechanisms.

## Results

### Bacterial Infection Promotes Phosphorylation of BCL2 and Induces Dissociation of the BCL2–BECN1 Complex in Small Intestinal Epithelial Cells.

**Fig. 1.**
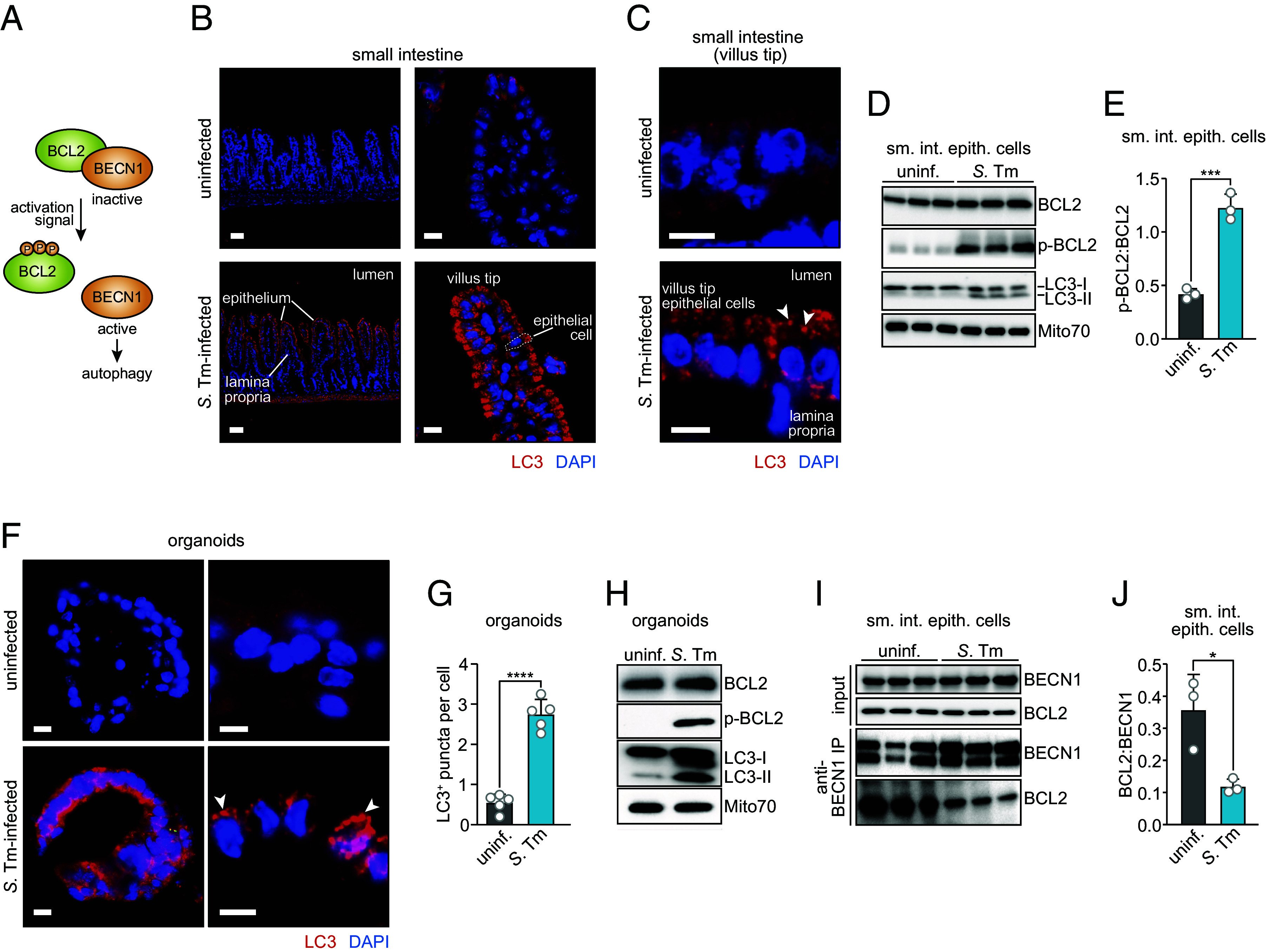
Bacterial infection promotes phosphorylation of BCL2 and induces dissociation of the BCL2–BECN1 complex in small intestinal epithelial cells. (*A*) Schematic of the BCL2–BECN1 interaction and its role in activating autophagy. BECN1 initiates autophagosome formation (9). BCL2 complexes with BECN1 to inhibit autophagy, and metabolic signals arising from starvation or exercise trigger dissociation of the BCL2–BECN1 complex as a prerequisite to autophagy (12, 13). The mechanism requires phosphorylation of BCL2, which induces dissociation from BECN1 and promotes autophagy (15). (*B*) Immunofluorescence microscopy of LC3 in the small intestines of uninfected and *S.* Typhimurium–infected conventional wild-type mice. Mice were infected intragastrically with 5 × 10^9^ colony-forming units (CFU) of *S.* Typhimurium (SL1344) and killed 24 h later. Sections of the distal small intestine were stained with rabbit anti-LC3 and Cy3-conjugated anti-rabbit IgG (red) and counterstained with DAPI (blue) to detect nuclei. [Scale bars, 50 μm (*Left*); 10 μm (*Right*)]. (*C*) Immunofluorescence microscopy of LC3 in the small intestinal villus tips of uninfected and *S.* Typhimurium–infected germ-free wild-type mice. Mice were infected and LC3^+^ structures were detected as in *B*. Examples of LC3^+^ puncta are indicated with arrowheads. (Scale bars, 10 μm.) Results are representative of images from n = 3 mice per group and five independent experiments. Immunofluorescence controls are shown in *SI Appendix*, Fig. S1. (*D*) Immunoblot analysis of small intestinal epithelial cells from uninfected or *S.* Typhimurium–infected germ-free wild-type mice. Mice were infected intragastrically as in *B*. Epithelial cell lysates were blotted and probed with antibodies against BCL2, p-BCL2, LC3, and Mito70 (loading control). LC3-I and LC3-II denote nonlipidated and lipidated LC3, respectively. Each lane represents one mouse. Results are representative of three independent experiments. (*E*) p-BCL2 and BCL2 band densities in *D* were measured by scanning densitometry and the ratio of the densities was calculated. Each data point represents one mouse. (*F*) Immunofluorescence microscopy of organoids derived from the small intestines of wild-type C57Bl/6 mice. Organoids were infected with 10^6^ CFU of *S.* Typhimurium (SL1344) for 4 h, then fixed, embedded, and sectioned. Sections were detected with rabbit anti-LC3 and Cy3-conjugated anti-rabbit IgG (red) and counterstained with DAPI (blue) to detect nuclei. Arrowheads indicate examples of LC3^+^ puncta. (Scale bars, 10 μm.) (*G*) LC3^+^ structures in images from *E* were counted. Each data point is the average number of LC3^+^ puncta in each cell of organoids from an individual mouse; at least 10 individual organoids were counted from each mouse (n = 3 mice per group). Results are representative of three independent experiments. (*H*) Immunoblot analysis of wild-type small intestinal organoids with and without *S.* Typhimurium infection. Organoids cultured from wild-type mice were infected with 10^6^ CFU of *S.* Typhimurium for 4 h. Organoid lysates were immunoblotted with antibodies against BCL2, p-BCL2, LC3, and Mito70 (loading control). LC3-I and LC3-II denote nonlipidated and lipidated LC3, respectively. Results are representative of three independent experiments. (*I*) Coimmunoprecipitation of BECN1 with BCL2 in small intestinal epithelial cells from uninfected or *S.* Typhimurium–infected germ-free mice. Epithelial cell lysates were immunoprecipitated with mouse anti-BECN1, blotted, and probed with mouse anti-BCL2-HRP plus rabbit anti-BECN1 (Novus Biologicals) and goat anti-rabbit IgG-HRP. The presence of two BECN1 isoforms in the immunoprecipitate has been observed in published reports (16–18). Each lane represents one mouse. Results are representative of three independent experiments. (*J*) Band intensities from the BECN1 immunoprecipitations in *I* were measured by scanning densitometry and the ratios of the BCL2 and BECN1 densities were calculated. Each data point represents one mouse. uninf., uninfected; *S.* Tm, *Salmonella* Typhimurium; sm. int. epith. cells, small intestinal epithelial cells; p-BCL2, phosphorylated BCL2; IP, immunoprecipitation. Means ± SEM are plotted; *P < 0.05; ***P < 0.001; ****P < 0.0001 by Student’s *t* test.

*S.* Typhimurium infection of the intestine activates epithelial cell autophagy which restricts intracellular bacteria (5, 6). To better understand the activation of antibacterial autophagy, we asked whether *S.* Typhimurium triggers molecular events like those involved in activating autophagy in response to metabolic signals. These events include phosphorylation of the autophagy inhibitor BCL2, which triggers dissociation of the BCL2–BECN1 complex and frees BECN1 to initiate the autophagy process (Fig. 1*A*).

LC3 is an essential autophagy protein that is recruited from the cytoplasm to the autophagosome membrane upon autophagy activation and can be detected in morphologically distinct structures by immunofluorescence (19, 20). We previously showed that *S.* Typhimurium activates autophagy in small intestinal enterocytes of both conventional and germ-free mice (5). Consistent with our prior findings (5), there were punctate LC3^+^ structures in the villus tips of small intestinal enterocytes 24 h after *S.* Typhimurium infection of germ-free mice, indicating autophagosome formation (Fig. 1*B* and *SI Appendix*, Fig. S1). During recruitment to the autophagosome, LC3-I is lipidated to yield LC3-II, which associates with the autophagosome membrane (21). Consistent with our prior findings (5), there was increased conversion of LC3-I to LC3-II in intestinal epithelial cells after *S.* Typhimurium infection, indicating autophagy activation (Fig. 1*C*). At the same time, while total BCL2 levels were similar between infected and uninfected mice, infected epithelial cells had more phosphorylated BCL2 (p-BCL2) (Fig. 1 *C* and *D*). Thus, *S.* Typhimurium infection promotes phosphorylation of BCL2 in small intestinal epithelial cells.

Small intestinal organoids that develop all epithelial cell lineages of the mouse intestinal epithelium can be established from adult Lgr5^+^ stem cells and expanded indefinitely in culture (22, 23). Since organoids are composed exclusively of epithelial cells, they can be used to decouple immune cell modulation of epithelial responses from epithelial cell-intrinsic processes. Small intestinal organoids derived from wild-type mice (*SI Appendix*, Fig. S2) were infected with *S.* Typhimurium or were left uninfected. LC3^+^ puncta were present in *S.* Typhimurium–infected organoids but were rare in uninfected organoids (Fig. 1 *E* and *F*). There was also more conversion of LC3-I to LC3-II in infected than in uninfected organoids (Fig. 1*G*), indicating autophagy activation. While total BCL2 protein levels were similar, there was more p-BCL2 in infected than uninfected organoids (Fig. 1*G*), confirming our findings in mice (Fig. 1 *C* and *D*). These results support our conclusion that *S.* Typhimurium induces BCL2 phosphorylation in intestinal epithelial cells and indicate that this process is intrinsic to epithelial cells.

During initiation of starvation-induced autophagy, phosphorylation of BCL2 triggers its dissociation from BECN1, freeing BECN1 to initiate the autophagy process (15). Since *S.* Typhimurium induced BCL2 phosphorylation, we tested for dissociation of the BCL2–BECN1 complex in infected small intestinal epithelial cells. Coimmunoprecipitation assays on mouse intestinal epithelial cell lysates showed reduced association of BCL2 with BECN1 following *S.* Typhimurium infection (Fig. 1 *H* and *I*). Thus, *S.* Typhimurium infection induces BCL2 phosphorylation and promotes dissociation of the inhibitory BCL2–BECN1 complex in small intestinal epithelial cells.

### BCL2 Phosphorylation Promotes Antibacterial Autophagy in Small Intestinal Epithelial Cells.

**Fig. 2.**
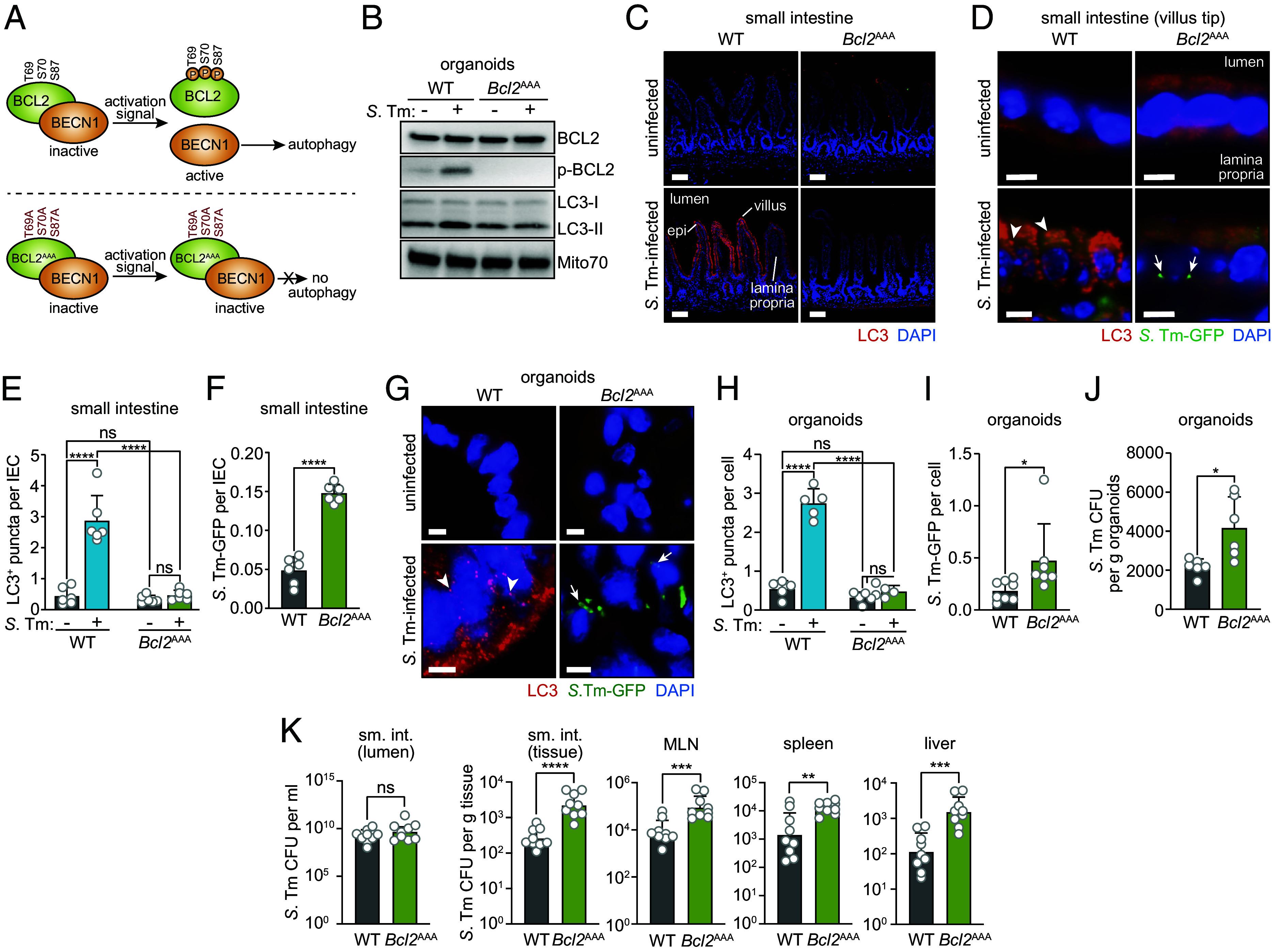
BCL2 phosphorylation promotes antibacterial autophagy in small intestinal epithelial cells. (*A*) Schematic depicting how the BCL2^AAA^ mutation impacts the BCL2–BECN1 interaction and autophagy activation. Activating signals promote BCL2 phosphorylation at three conserved amino acid residues: T69, S70, and S87 (15). *Bcl2*^AAA^ mice harbor a knock-in *Bcl2* allele in which these residues are mutated, rendering BCL2 nonphosphorylatable and unable to release BECN1 to initiate autophagy. These mice are thus deficient in stimulus-induced but not basal autophagy (13). (*B*) Immunoblot of small intestinal organoids from wild-type (WT) and *Bcl2*^AAA^ mice. Organoids were infected with *S.* Typhimurium–GFP (10^6^ CFU) for 4 h or were left uninfected. Immunoblots were detected with antibodies against BCL2, p-BCL2, LC3, and Mito70 (loading control). LC3-I and LC3-II denote nonlipidated and lipidated LC3, respectively. Results are representative of three independent experiments. (*C*) Immunofluorescence microscopy of small intestines from infected and uninfected conventionally raised wild-type and *Bcl2*^AAA^ littermates. Mice were infected intragastrically with *S.* Typhimurium–GFP (5 × 10^9^ CFU per mouse) and were killed 24 h later. Sections of the distal small intestine were stained with rabbit anti-LC3 and Cy3-conjugated anti-rabbit IgG (red), and goat anti-GFP and DyLight 488-conjugated anti-goat IgG (green). Intrinsic GFP fluorescence is destroyed by tissue fixation, necessitating antibody detection of GFP. Sections were counterstained with DAPI (blue) to detect nuclei. (Scale bars, 50 μm.) (*D*) Immunofluorescence microscopy of small intestinal villus tips from uninfected and *S.* Typhimurium–infected mice. Mice were infected and tissues stained as in *C*. Examples of LC3^+^ structures are indicated with arrowheads, and examples of intracellular *S.* Typhimurium–GFP are indicated with arrows. (Scale bars, 10 μm.) Images are representative n = 6 mice per group and two independent experiments. Immunofluorescence controls are shown in *SI Appendix*, Fig. S1. (*E*) LC3^+^ puncta in images from *D* were counted. Each data point is the average number of LC3^+^ puncta per cell from one mouse (n = 6 mice per group). At least 200 crypt-villus units were counted per mouse, and all epithelial cells in each crypt-villus unit were counted (note that enterocytes at the villus tips typically have more LC3^+^ puncta than enterocytes in the lower regions of the crypt-villus unit) (5). (*F*) Cell-associated *S.* Typhimurium–GFP in images from *D* were counted. Each data point is the average number of *S.* Typhimurium per intestinal epithelial cell (IEC) from one mouse (n = 6 mice per group); at least 200 crypt-villus units were counted per mouse. (*G*) Immunofluorescence microscopy of small intestinal organoids grown from wild-type and *Bcl2*^AAA^ littermates. Organoids were infected with 10^6^ CFU of *S.* Typhimurium–GFP for 4 h, then fixed, embedded, and sectioned. Sections were detected with rabbit anti-LC3 and Cy3-conjugated anti-rabbit IgG (red), and goat anti-GFP, DyLight 488-conjugated anti-goat IgG (green), and DAPI (blue) to detect nuclei. Arrowheads indicate examples of LC3^+^ puncta. (Scale bars, 5 μm.) Images are representative of organoids from n = 5 mice per group and two independent experiments. (*H*) LC3^+^ puncta in images from *G* were counted. Each point is the average number of LC3^+^ puncta in each organoid cell from one mouse (n = 5 mice per group); at least 10 organoids were counted from each mouse. (*I*) Intracellular *S.* Typhimurium–GFP in images from *G* were counted. Each point represents organoids from one mouse (n = 8 mice per group and three independent experiments). (*J*) Numbers of intracellular *S.* Typhimurium–GFP in organoids grown from wild-type and *Bcl2*^AAA^ littermates as determined by dilution plating. Each point represents organoids from one mouse (n = 6 mice per group). (*K*) Bacterial burdens CFU in the small intestine (sm. int.), mesenteric lymph node (MLN), spleen, and liver of wild-type and *Bcl2*^AAA^ littermates 24 h after intragastric infection with 5 × 10^9^ CFU *S.* Typhimurium–GFP. Bacterial counts were determined by dilution plating. Each point represents an individual mouse, and data are from three independent experiments. Geometric means ± SEM are plotted. WT, wild-type; *S.* Tm, *Salmonella* Typhimurium; GFP, green fluorescent protein; IEC, intestinal epithelial cell; p-BCL2, phosphorylated BCL2; CFU, colony-forming units. Means ± SEM are plotted except where noted; *P < 0.05; **P < 0.01; ****P < 0.0001; ns, not significant by Student’s *t* test.

Because *S.* Typhimurium induced phosphorylation of BCL2 and triggered its dissociation from BECN1, we hypothesized that BCL2 phosphorylation promotes antibacterial autophagy in enterocytes. To test this idea, we studied mice with a knock-in allele of *Bcl2* in which the three conserved phosphorylation sites (Thr69, Ser70, and Ser84) are mutated to alanine (*Bcl2*^AAA^ mice) (13) (Fig. 2*A*). Phosphorylation of the mutant BCL2 protein (BCL2^AAA^) is blocked and thus the mutant BCL2 cannot dissociate from BECN1, keeping BECN1 in the inhibited state (13) (Fig. 2*A*). The *Bcl2*^AAA^ mice are therefore deficient in stimulus-induced but not basal autophagy (13). The *Bcl2*^AAA^ mutation specifically impacts the anti-autophagy function of BCL2 and does not inhibit its antiapoptotic function in tissues including the intestinal epithelium (13) (*SI Appendix*, Fig. S3).

To confirm that *Bcl2*^AAA^ intestinal epithelial cells show defective BCL2 phosphorylation, we infected organoids derived from wild-type and *Bcl2*^AAA^ mice with *S.* Typhimurium and assessed BCL2 phosphorylation by immunoblot. While total BCL2 levels were similar in wild-type and *Bcl2*^AAA^ organoids, p-BCL2 was present in wild-type organoids but was mostly absent in the *Bcl2*^AAA^ organoids (Fig. 2*B*). Thus, *Bcl2*^AAA^ epithelial cells are unable to phosphorylate BCL2 in response to bacterial infection.

We next tested whether bacterial infection can induce autophagy in the enterocytes of *Bcl2*^AAA^ mice. We infected wild-type and *Bcl2*^AAA^ mice intragastrically with *S.* Typhimurium expressing green fluorescent protein (*S.* Typhimurium–GFP) so that we could visualize the bacteria within cells. At 24 h after infection, there were fewer LC3^+^ puncta in enterocytes from *Bcl2*^AAA^ mice as compared to wild-type mice (Fig. 2 *C* and *D*). Consistent with the rapid elimination of intracellular bacteria by autophagy (5), we observed small numbers of *S.* Typhimurium–GFP within the enterocytes of wild-type mice, with higher numbers of *S.* Typhimurium–GFP present in enterocytes from *Bcl2*^AAA^ mice (Fig. 2 *C* and *E*). This indicates that autophagy activation and clearance of intracellular bacteria are impaired in enterocytes of *Bcl2*^AAA^ mice.

The *Bcl2*^AAA^ mice harbor the mutation in all cells, raising the possibility that the requirement for BCL2 phosphorylation in epithelial autophagy originated in a cell of nonepithelial lineage. To determine whether the requirement for BCL2 phosphorylation is intrinsic to epithelial cells, we assessed autophagy activation in *S.* Typhimurium–infected organoids derived from *Bcl2*^AAA^ mice. In accordance with our findings in mice, there was less conversion of LC3-I to LC3-II (Fig. 2*B*), fewer LC3^+^ puncta (Fig. 2 *F* and *G*), and more intracellular bacteria (Fig. 2 *H* and *I*) in *Bcl2*^AAA^ organoids than wild-type organoids. Because organoids are strictly of epithelial cell origin (23), this suggests that the requirement for BCL2 phosphorylation in antibacterial autophagy is intrinsic to epithelial cells. Taken together, our findings in mice and organoids show that BCL2 phosphorylation is required for bacterial induction of autophagy and restricts intracellular bacteria in intestinal epithelial cells.

We previously found that intestinal epithelial autophagy promotes host resistance to *S.* Typhimurium infection and dissemination (5). Because *Bcl2*^AAA^ mice had impaired enterocyte autophagy in response to bacterial infection, we predicted that these mice would show decreased resistance to *S.* Typhimurium infection. After a 24-h oral infection with *S.* Typhimurium, bacterial numbers in the small intestinal lumen were not significantly different between wild-type and *Bcl2*^AAA^ mice (Fig. 2*J*). However, more bacteria were recovered from small intestinal tissue, mesenteric lymph nodes (MLN), spleen, and liver in *Bcl2*^AAA^ mice (Fig. 2*J*). The *Bcl2*^AAA^ mice did not show increased permeability to orally administered fluorescein isothiocyanate (FITC)-dextran (*SI Appendix*, Fig. S4), indicating that the increased bacterial dissemination was not due to enhanced nonspecific barrier permeability. Although these results indicate that BCL2 phosphorylation limits *S.* Typhimurium dissemination in mice, the presence of the *Bcl2*^AAA^ mutation in all cells means that we cannot exclude the possibility that BCL2 phosphorylation in nonepithelial cells contributes to the increased bacteria burden. We also cannot rule out that phosphorylation of BCL2 promotes infection resistance by impacting cellular functions other than canonical autophagy. These limitations are further considered in the *Discussion*.

### JNK1 Phosphorylates BCL2 and Regulates Antibacterial Autophagy in Small Intestinal Enterocytes.

We next sought to clarify the enzymatic pathway of invasion-induced BCL2 phosphorylation in intestinal epithelial cells. In starvation-induced autophagy, the kinase JNK1 phosphorylates BCL2, leading to the disruption of the BCL2–BECN1 complex and autophagy activation (15). We therefore assessed whether JNK1 regulates BCL2 phosphorylation in infected intestinal epithelial cells.

**Fig. 3.**
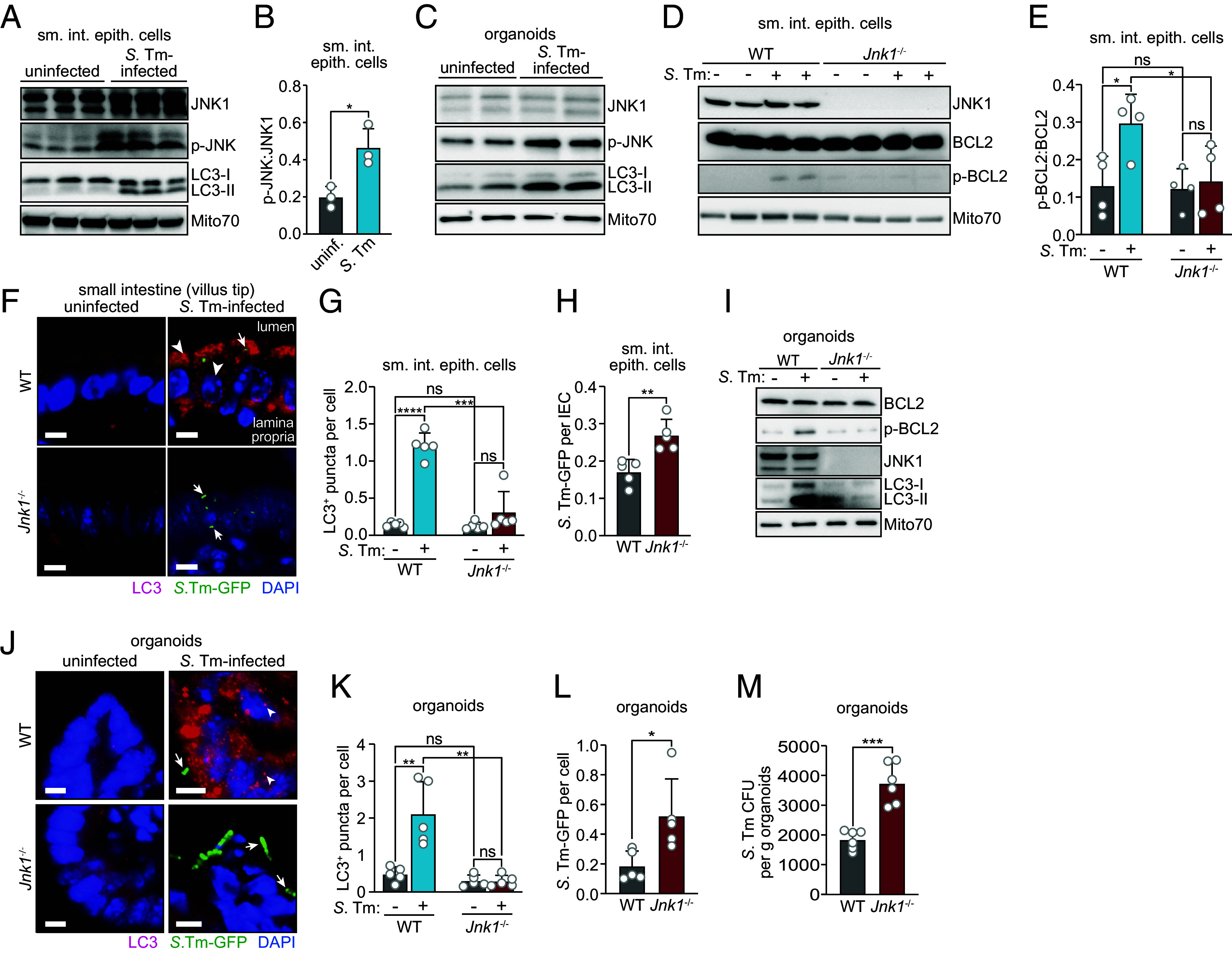
JNK1 phosphorylates BCL2 and regulates antibacterial autophagy in small intestinal enterocytes. (*A*) Immunoblot of small intestinal epithelial cells from uninfected or *S.* Typhimurium–infected germ-free wild-type mice. Mice were infected intragastrically with 5 × 10^9^ CFU of *S.* Typhimurium–GFP and were killed 24 h later. Small intestinal epithelial cell lysates were blotted and probed with antibodies against JNK1, p-JNK, LC3, and Mito70 as a loading control. LC3-I and LC3-II denote nonlipidated and lipidated LC3, respectively. Each lane represents one mouse. Results are representative of three independent experiments. (*B*) The p-JNK and JNK1 band intensities in *A* were measured by scanning densitometry and the ratio of the densities was calculated. Each data point represents one mouse. (*C*) Immunoblot of wild-type small intestinal organoids with and without *S.* Typhimurium infection. Organoids were infected with 10^6^ CFU of *S.* Typhimurium–GFP for 4 h. Organoid lysates were immunoblotted with antibodies against JNK1, p-JNK, LC3, and Mito70 (loading control). Results are representative of three independent experiments. (*D*) Immunoblot of small intestinal epithelial cells from conventional wild-type and *Jnk1*^−/−^ mice. Mice were infected intragastrically with *S.* Typhimurium–GFP (5 × 10^9^ CFU per mouse) and were killed 24 h later. JNK1, BCL2, p-BCL2, and Mito70 (loading control) were detected. Each lane represents one mouse. Results are representative of three independent experiments. (*E*) The p-BCL2 and BCL2 band intensities in *D* were measured by scanning densitometry and the ratio of the densities was calculated. Each data point represents one mouse (n = 4 mice per group). (*F*) Immunofluorescence microscopy of small intestines from *S.* Typhimurium–infected conventional wild-type and *Jnk1*^−/−^ mice. Wild-type and *Jnk1*^−/−^ littermates were orally infected with *S.* Typhimurium–GFP (5 × 10^9^ CFU per mouse) and were killed 24 h later. Sections of the distal small intestine were stained with rabbit anti-LC3 and Cy3-conjugated anti-rabbit IgG (red), and goat anti-GFP and DyLight 488-conjugated anti-goat IgG (green). Sections were counterstained with DAPI (blue) to detect nuclei. Examples of LC3^+^ structures are indicated with arrowheads, and examples of intracellular *S.* Typhimurium–GFP are indicated with arrows. (Scale bars, 5 μm.) Images are representative of n = 5 mice per group and two independent experiments. (*G*) LC3^+^ puncta from images in *F* were counted. Each data point represents the average number of LC3^+^ puncta per cell from one mouse; at least 200 villi were counted per mouse (n = 5 mice per group). (*H*) Epithelial cell-associated *S.* Typhimurium–GFP from images in *F* were counted. Each data point represents the average number of *S.* Typhimurium per IEC from one mouse; at least 200 villi were counted per mouse (n = 5 mice per group). (*I*) Immunoblot of small intestinal organoids from wild-type and *Jnk1*^−/−^ mice. Organoids were infected with 10^6^ CFU of *S.* Typhimurium–GFP for 4 h, then embedded and sectioned. BCL2, p-BCL2, JNK1, LC3, and Mito70 (loading control) were detected with antibodies as in *A*. Results are representative of three independent experiments. (*J*) Immunofluorescence microscopy of small intestinal organoids from wild-type and *Jnk1*^−/−^ mice. Organoids were fixed, embedded, and sectioned. Sections were detected with rabbit anti-LC3 and Cy3-conjugated anti-rabbit IgG (red), and goat anti-GFP, DyLight 488-conjugated anti-goat IgG (green), and DAPI (blue) to detect nuclei. Arrowheads indicate examples of LC3^+^ puncta. (Scale bars, 5 μm.) Images are representative of organoids from n = 3 mice per group and two independent experiments. (*K*) LC3^+^ puncta in images from *J* were counted. Each data point is the average number of LC3^+^ puncta in each cell; at least 10 organoids were counted from each mouse. (*L*) Intracellular *S.* Typhimurium–GFP in images from *J* were counted. Each data point represents organoids from one mouse (n = 5 mice per group). (*M*) Numbers of intracellular *S.* Typhimurium–GFP in organoids grown from wild-type and *Jnk1*^−/−^ mice as determined by dilution plating. Each data point represents organoids from one mouse (n = 6 mice per group). sm. int. epith. cells, small intestinal epithelial cells; uninf., uninfected; *S*. Tm, *Salmonella* Typhimurium; p-JNK, phosphorylated JNK; p-BCL2, phosphorylated BCL2; IEC, intestinal epithelial cell; GFP, green fluorescent protein; WT, wild-type. Means ± SEM are plotted; *P < 0.05; **P < 0.01; ***P < 0.001; ****P < 0.0001; ns, not significant by Student’s *t* test.

First, we evaluated whether *S.* Typhimurium JNK1 is activated in intestinal epithelial cells following *S.* Typhimurium infection. We performed immunoblots on epithelial cells isolated from wild-type mice with and without *S.* Typhimurium infection to detect phosphorylated JNK (p-JNK), the activated form of JNK (15). While total levels of JNK1 were similar, there was more p-JNK in epithelial cells from infected than uninfected mice (Fig. 3 *A* and *B*). Similarly, p-JNK levels increased upon infection of small intestinal organoids (Fig. 3*C*), supporting our findings in mice. The increase in p-JNK coincided with increased p-BCL2 (Fig. 3 *D* and *E*) and increased conversion of LC3-I to LC3-II (Fig. 3 *A* and *C*). Thus, *S.* Typhimurium infection activates JNK1 in intestinal epithelial cells.

We next tested whether JNK1 is necessary for BCL2 phosphorylation and autophagy activation in epithelial cells by studying *Jnk1*^−/−^ mice. Although total BCL2 levels were similar between infected wild-type and *Jnk1*^−/−^ mice, epithelial cells from infected *Jnk1*^−/−^ mice had less p-BCL2 (Fig. 3 *D* and *E*). There were fewer LC3^+^ puncta and more *S.* Typhimurium–GFP in epithelial cells from infected *Jnk1*^−/−^ mice as compared to wild-type mice (Fig. 3*F* through Fig. 3*H*), indicating that *Jnk1*^−/−^ mice are resistant to epithelial autophagy activation. Similarly, there was less p-BCL2, fewer LC3^+^ puncta, and more intracellular bacteria in infected organoids derived from *Jnk1*^−/−^ mice than wild-type mice (Fig. 3*I* through Fig. 3*M*), indicating that the requirement for JNK1 is intrinsic to epithelial cells. Thus, JNK1 is necessary for BCL2 phosphorylation and autophagy activation in infected intestinal epithelial cells.

### Constitutively Active BECN1 Rescues the Autophagy Block in MYD88-Deficient Enterocytes.

Activation of antibacterial autophagy in enterocytes requires the TLR signaling adaptor MYD88. Bacterial induction of autophagy is blocked in MYD88-deficient mice, and the requirement for MYD88 is intrinsic to epithelial cells (5). However, it is unclear how MYD88 signaling directs autophagy initiation. We considered whether MYD88 might regulate BCL2 phosphorylation and thus dissociation of the inhibitory BCL2–BECN1 complex. This possibility is suggested by studies showing that c-Jun N-terminal protein kinases, including JNK1, are activated by MYD88 signaling to control innate immunity to infection (24, 25). An alternative possibility is that MYD88 signaling regulates events downstream of BCL2–BECN1 dissociation that are also required for autophagy activation.

**Fig. 4.**
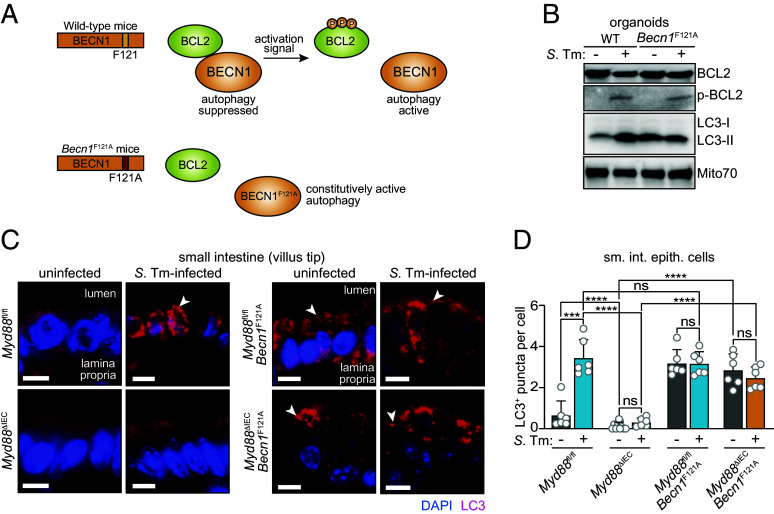
Constitutively active BECN1 rescues the autophagy block in MYD88-deficient enterocytes. (*A*) BECN1^F121A^ harbors a Phe-to-Ala point mutation that disrupts the interaction of BECN1 with BCL2, yielding BECN1 that is constitutively active (12, 26, 27). *Becn1*^F121A^ knock-in mice thus have increased basal levels of autophagy (12). (*B*) Immunoblot analysis of BCL2 phosophorylation in wild-type and *Becn1*^F121A^ small intestinal organoids with and without *S.* Typhimurium infection. Organoids were infected with 10^6^ CFU of *S.* Typhimurium–GFP for 4 h. Organoid lysates were immunoblotted and detected with antibodies against BCL2, p-BCL2, LC3, and Mito70 (loading control). Results are representative of three independent experiments. (*C*) Immunofluorescence microscopy of small intestinal villus in *Myd88*^fl/fl^, *Myd88*^ΔIEC^, *MyD88*^fl/fl^/*Becn1*^F121A^ and MyD88^ΔIEC^/*Becn1*^F121A^ mice. Mice were infected intragastrically with *S.* Typhimurium–GFP (5 × 10^9^ CFU per mouse) and killed 24 h later. Sections of the distal small intestine were stained with rabbit anti-LC3 and Cy3-conjugated anti-rabbit IgG (red). Sections were counterstained with DAPI (blue) to detect nuclei. Examples of LC3^+^ puncta are indicated with arrowheads. (Scale bars, 10 μm.) Images are representative of n = 3 mice per group and two independent experiments. (*D*) LC3^+^ puncta from images in *C* were counted. Each data point represents the average number of LC3^+^ puncta per cell from one mouse; at least 200 crypt-villus units were counted per mouse (n = 6 mice per group). *S*. Tm, *Salmonella* Typhimurium; WT, wild-type; p-BCL2, phosphorylated BCL2; sm. int. epith. cells, small intestinal epithelial cells. Means ± SEM are plotted; ***P < 0.001; ****P < 0.0001; ns, not significant by Student’s *t* test.

To distinguish between these possibilities, we studied mice harboring a Phe-to-Ala knock-in substitution mutation in BECN1 (F121A) (26, 27). This point mutation restricts BCL2 binding to BECN1, leaving BECN1 free to initiate autophagy regardless of BCL2 phosphorylation (Fig. 4*A*) (12, 27). If MYD88 signaling directs BCL2 phosphorylation, the *Becn1*^F121A^ mutation should rescue the autophagy defect in mice with an epithelial cell-specific deletion of *Myd88* (*Myd88*^ΔIEC^ mice) (5). Conversely, if MYD88 regulates autophagy by controlling events downstream of BCL2 phosphorylation, then the *Becn1*^F121A^ mutation should fail to rescue the autophagy defect in *Myd88*^ΔIEC^ mice.

We first confirmed that BCL2 is phosphorylated normally in infected *Becn1*^F121A^ epithelial cells. We infected organoids derived from wild-type and *Becn1*^F121A^ mice with *S.* Typhimurium and assessed BCL2 phosphorylation by immunoblot. As expected, total levels of BCL2 were similar and p-BCL2 levels increased upon infection of both wild-type and *Becn1*^F121A^ organoids (Fig. 4*B*). Further, uninfected and infected *Becn1*^F121A^ organoids both showed increased conversion of LC3-I to LC3-II relative to uninfected wild-type organoids, indicating constitutive autophagy that does not require an activation signal (Fig. 4*B*). This indicates that *S.* Typhimurium activates the pathway upstream of BECN1 in *Becn1*^F121A^ epithelial cells and that constitutive autophagy is independent of infection. Although we predicted that the increased constitutive autophagy in the *Becn1*^F121A^ mice would enhance clearance of *S.* Typhimurium from intestinal tissue, we did not detect a significant difference in bacterial numbers in the intestinal lumen or tissue when comparing infected wild-type and *Becn1*^F121A^ mice (*SI Appendix*, Fig. S5). We suggest that this may be because ongoing constitutive autophagy overconsumes cellular resources, such as membranes, required for the formation of new autophagosomes around invading bacteria.

We next intercrossed *Myd88*^ΔIEC^ and *Becn1*^F121A^ mice and infected the resulting *Myd88*^ΔIEC^/*Becn1*^F121A^ mice. As we observed previously (5), there were fewer LC3^+^ puncta in epithelial cells from *S.* Typhimurium–infected *Myd88*^ΔIEC^ mice as compared to infected *Myd88*^fl/fl^ mice (Fig. 4 *C* and *D*). However, the presence of the *Becn1*^F121A^ allele increased the numbers of LC3^+^ puncta in both uninfected and infected *Myd88*^fl/fl^ and *Myd88*^ΔIEC^ mice (Fig. 4 *C* and *D*). Thus, constitutively active BECN1 rescues the autophagy block in MYD88-deficient intestinal epithelial cells, indicating that BECN1 is activated downstream of MYD88.

### MYD88 Controls JNK1 and BCL2 Phosphorylation and Dissociation of the BCL2–BECN1 Complex in Small Intestinal Epithelial Cells.

**Fig. 5.**
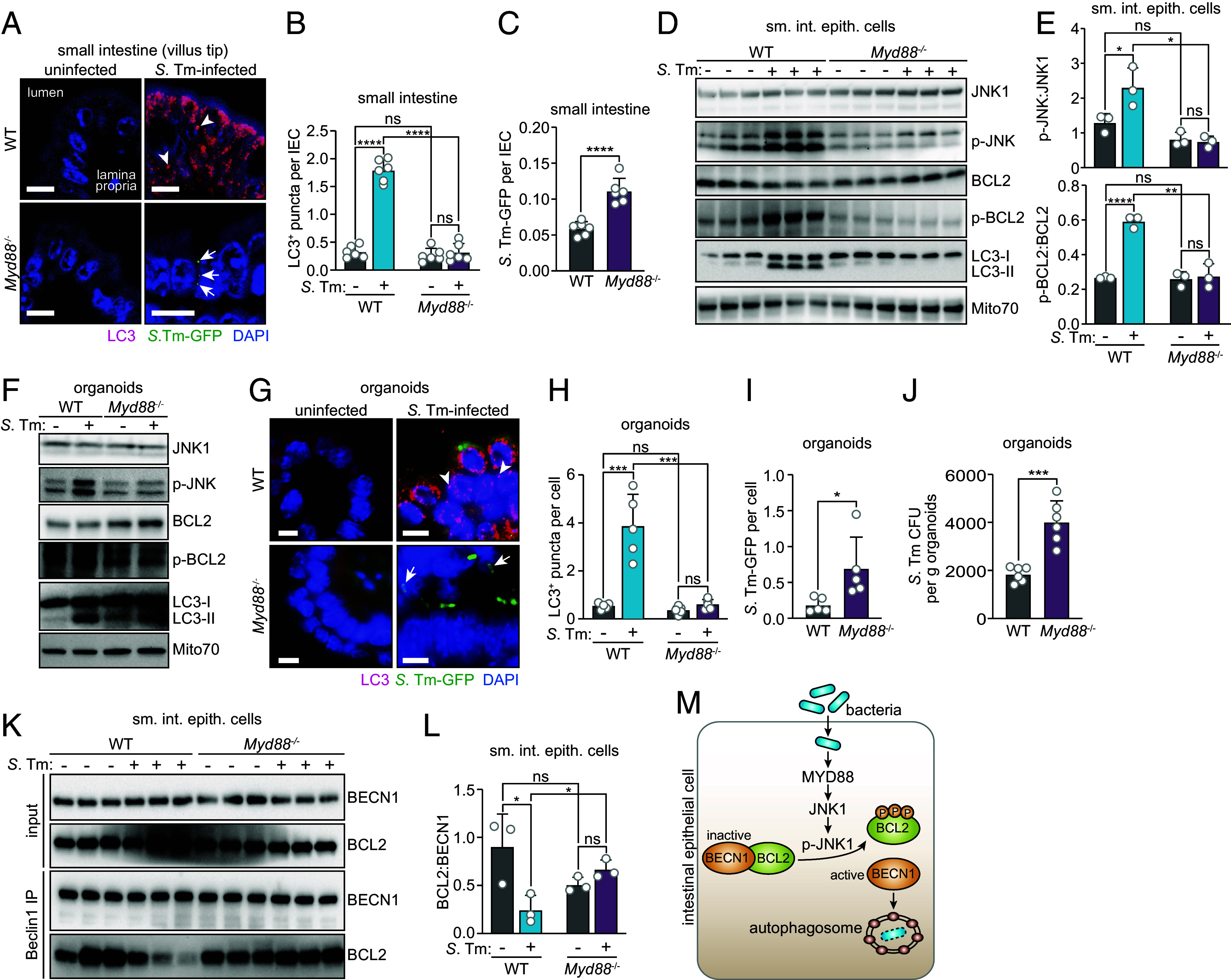
MYD88 controls JNK1 and BCL2 phosphorylation and dissociation of the BCL2–BECN1 complex in small intestinal epithelial cells. (*A*) Immunofluorescence microscopy of small intestinal villus tips from uninfected or *S.* Typhimurium–infected germ-free wild-type or *Myd88*^−/−^ mice. Mice were infected intragastrically with *S.* Typhimurium–GFP (5 × 10^9^ CFU per mouse) and were killed 24 h later. Sections of the distal small intestine were stained with rabbit anti-LC3 and Cy3-conjugated anti-rabbit IgG (red), and goat anti-GFP and DyLight 488-conjugated anti-goat IgG (green). Sections were counterstained with DAPI (blue) to detect nuclei. Examples of LC3^+^ puncta are indicated with arrowheads, and examples of intracellular *S.* Typhimurium–GFP are indicated with arrows. (Scale bars, 10 μm.) Images are representative of n = 3 mice per group and two independent experiments. (*B*) LC3^+^ puncta in images from *A* were counted. Each data point is the average number of LC3^+^ puncta per cell from one mouse (n = 6 mice per group). At least 200 crypt-villus units were counted per mouse, and all cells in each crypt-villus unit were counted. (*C*) Epithelial cell-associated *S.* Typhimurium–GFP in images from *A* were counted. Each data point is the average number of *S.* Typhimurium per IEC from one mouse (n = 6 mice per group); at least 200 crypt-villus units were counted per mouse, and all cells in each crypt-villus unit were counted. (*D*) Immunoblot analysis of small intestinal epithelial cells from uninfected or *S.* Typhimurium–infected germ-free wild-type and *Myd88*^−/−^ mice. Mice were infected intragastrically with *S.* Typhimurium–GFP and were killed 24 h later. Small intestinal IEC lysates were blotted and probed with antibodies against JNK1, p-JNK, BCL2, p-BCL2, LC3, and Mito70 (loading control). LC3-I and LC3-II denote nonlipidated and lipidated LC3, respectively. Each lane represents one mouse. Results are representative of three independent experiments. (*E*) The band intensities in *D* were measured by scanning densitometry and the ratios of the p-JNK and JNK1 densities (*Top*) and p-BCL2 and BCL2 densities (*Bottom*) were calculated. Each data point represents one mouse. (*F*) Immunoblot analysis of wild-type and *Myd88*^−/−^ small intestinal organoids with and without *S.* Typhimurium infection. Organoids were infected with 10^6^ CFU of *S.* Typhimurium–GFP for 4 h. Organoid lysates were immunoblotted with antibodies against JNK1, p-JNK, BCL2, p-BCL2, LC3, and Mito70 (loading control). Results are representative of three independent experiments. (*G*) Immunofluorescence microscopy of wild-type and *Myd88*^−/−^ organoids. Organoids were infected with 10^6^ CFU of *S.* Typhimurium–GFP for 4 h, then fixed, embedded, and sectioned. Sections were detected with rabbit anti-LC3 and Cy3-conjugated anti-rabbit IgG (red), and goat anti-GFP and DyLight 488-conjugated anti-goat IgG (green). Sections were counterstained with DAPI (blue) to detect nuclei. Arrowheads indicate examples of LC3^+^ puncta, and arrows indicate examples of *S.* Typhimurium–GFP. (Scale bars, 5 μm.) Results are representative of n = 9 mice and three independent experiments. (*H*) LC3^+^ puncta in images from *G* were counted. Each data point is the average number of LC3^+^ puncta in each cell of organoids from an individual mouse; at least 10 organoids were counted from each mouse (n = 5 mice per group). Results are representative of two independent experiments. (*I*) Cell-associated *S.* Typhimurium–GFP in images from *G* were counted. Each data point is the average number of *S. Typhimurium* per IEC from one mouse (n = 5 mice per group). At least 200 crypt-villus units were counted per mouse, and all cells in each crypt-villus unit were counted. (*J*) Numbers of intracellular *S.* Typhimurium in organoids grown from wild-type and *Myd88^−/−^* littermates as determined by dilution plating. Each data point represents organoids from one mouse (n = 6 mice per group). (*K*) Coimmunoprecipitation of BECN1 with BCL2 in small intestinal epithelial cells from uninfected or *S.* Typhimurium–GFP–infected germ-free wild-type and *Myd88*^−/−^ mice. Epithelial cell lysates were immunoprecipitated with mouse anti-BECN1, blotted, and probed with mouse anti-BCL2-HRP plus rabbit anti-BECN1 (Santa Cruz) and goat anti-rabbit IgG HRP. Each lane represents one mouse. Results are representative of three independent experiments. (*L*) The band intensities in *K* were measured by scanning densitometry and the ratios of the BCL2 and BECN1 densities were calculated. Each data point represents one mouse. (*M*) Model: Bacterial infection of intestinal enterocytes induces phosphorylation of JNK1 downstream of MYD88. JNK1 phosphorylates BCL2, which dissociates from BECN1 and releases BECN1 to initiate autophagy. BCL2 thus regulates antibacterial autophagy in enterocytes to limit bacterial infection of the intestinal epithelial barrier. *S.* Tm, *Salmonella* Typhimurium; WT, wild-type; sm. int. epith. cells, small intestinal epithelial cells; IEC, intestinal epithelial cell; p-JNK, phosphorylated JNK; p-BCL2, phosphorylated BCL2; IP, immunoprecipitation. Means ± SEM are plotted; *P < 0.05; **P < 0.01; ***P < 0.001; ****P < 0.0001; ns, not significant by Student’s *t* test.

Because constitutively active BECN1 rescued the autophagy block arising from MYD88 deficiency, we postulated that MYD88 controls JNK1 activation and thus BCL2 phosphorylation. We tested this idea by infecting germ-free *Myd88^−/−^* mice with *S.* Typhimurium. As we previously reported (5), enterocytes of *Myd88^−/−^* mice were resistant to autophagy activation by *S.* Typhimurium, as evidenced by fewer LC3^+^ puncta (Fig. 5*A*), reduced conversion of LC3-I to LC3-II (Fig. 5*B*) and more intracellular bacteria (Fig. 5*C*). At the same time, there was less activation of JNK1 to p-JNK in *Myd88^−/−^* mice than in wild-type mice (Fig. 5 *D* and *E*). Consistent with the reduced JNK1 activation, the infected *Myd88^−/−^* mice had less p-BCL2 relative to infected wild-type mice (Fig. 5 *D* and *E*). There was also less JNK activation, less BCL2 phosphorylation, fewer LC3^+^ puncta, and fewer intracellular bacteria in *S.* Typhimurium–infected epithelial organoids derived from *Myd88^−/−^* mice (Fig. 5*F* through Fig. 5*J*), indicating that the requirement for MYD88 is intrinsic to epithelial cells. Thus, MYD88 controls JNK activation and BCL2 phosphorylation in infected intestinal epithelial cells.

Finally, we evaluated whether MYD88 regulates dissociation of the BCL2–BECN1 complex. Immunoprecipitation of small intestinal epithelial cell lysates with anti-BECN1 antibody showed that BCL2 remained associated with BECN1 following *S.* Typhimurium infection of germ-free *Myd88^−/−^* mice (Fig. 5 *K* and *L*). This contrasted with the reduced association of BCL2 with BECN1 after infection of germ-free wild-type mice (Fig. 5 *K* and *L*). These results indicate that MYD88 promotes dissociation of the BCL2–BECN1 complex in response to bacterial invasion. Together, our findings reveal that MYD88 stimulates BCL2 phosphorylation in gut epithelial cells, thus triggering dissociation of the BCL2–BECN1 complex and liberating BECN1 to initiate autophagy during bacterial invasion (Fig. 5*M*).

## Discussion

The intestinal epithelium plays a central role in defending against invasive intestinal bacteria. First, epithelial cells secrete mucin glycoproteins that form the mucus layer, which is a physical barrier that limits bacterial access to epithelial cells (28–30). Second, intestinal epithelial cells secrete antimicrobial proteins into the mucus layer that limit bacterial colonization of the mucus and attachment to the intestinal epithelial surface (29, 31–33). Epithelial cells also have mechanisms for eliminating bacteria that invade the cytoplasm after evading the mucus and antimicrobial barriers. One such mechanism is autophagy, which is activated in enterocytes by invasive bacteria such as *S.* Typhimurium (5, 6). Bacterial entry into the epithelial cell cytoplasm activates autophagosome formation, which clears intracellular bacteria and limits their dissemination to extraintestinal tissues (5).

Our findings from this study provide molecular insight into how innate immune signaling activates antibacterial autophagy in intestinal epithelial cells. Our previous work showed that bacterial activation of autophagy in enterocytes requires epithelial cell MYD88 (5). However, it was unclear how MYD88 signaling connects to autophagy pathways to regulate autophagy initiation. Here, we have found that MYD88 signaling promotes autophagy by triggering dissociation of the inhibitory BCL2–BECN1 complex (Fig. 5*M*). *S.* Typhimurium stimulates MYD88-dependent activation of the downstream kinase JNK1. Activated JNK1 phosphorylates BCL2, causing it to dissociate from BECN1, which liberates BECN1 to initiate autophagy and restrict intracellular bacteria.

A central insight from our findings is the identification of BCL2 as a molecular link between MYD88 signaling and autophagosome nucleation. BCL2 was originally described as having antiapoptotic activity stemming from its ability to antagonize the proapoptotic proteins, BAX and BAK (34). Subsequently, BECN1 was identified as a BCL2-interacting protein that is essential for autophagy (9, 35). BCL2 inhibits autophagy by binding to BECN1 (12), and this interaction is regulated by metabolic stressors such as starvation and exercise (12, 13). Further, BCL2 and its interaction with BECN1 regulate the clearance of amyloid β oligomers that contribute to a mouse model of Alzheimer’s disease (26), and impact mouse lifespan (27). Our results show that BCL2 regulates antibacterial autophagy through the same BCL2 phosphorylation pathway that is activated in metabolic autophagy. Our findings thus expand the understanding of the cellular functions of BCL2 and reveal that metabolic and microbial stimuli can activate autophagy through a common BCL2-dependent mechanism.

Several important questions remain about the epithelial cell MYD88-JNK-BCL2 pathway. First, the host receptors that lie upstream of this pathway remain to be defined. Our prior finding that autophagy activation requires invasive bacteria (5) raises the possibility that endosomal TLRs, such as TLR9, could be involved in the recognition of invading bacteria that trigger autophagy. However, studies in TLR reporter mice suggest a very low level of TLR9 expression in the small intestinal epithelium, thus arguing against a prominent role for TLR9 in epithelial autophagy activation (36). Alternatively, invading bacteria could activate basolateral TLRs, such as TLR5 (37), by traversing the epithelial barrier either through a paracellular route or by exiting epithelial cells via their basolateral surface. This idea is supported by reporter mouse studies showing prominent TLR5 expression in epithelial cells of the small intestine (36). Another possibility is that MYD88-dependent autophagy occurs downstream of IL-1 or IL-18 receptors, which also signal through MYD88 (24, 38). Second, it remains unclear how MYD88-dependent regulation of the BCL2–BECN1 complex interacts with the pathways that govern recruitment of the autophagy machinery to the bacterial surface (2, 3).

Our experiments in *Bcl2*^AAA^ mice show that BCL2 phosphorylation limits *S.* Typhimurium dissemination. However, the *Bcl2*^AAA^ mutation is not restricted to epithelial cells in these mice, thus limiting our interpretation of these studies. Although our *Bcl2*^AAA^ organoid experiments indicate an epithelial cell autonomous role for BCL2 in restricting bacteria, it is possible that cells other than enterocytes may contribute to the decreased infection resistance in *Bcl2*^AAA^ mice. For example, autophagy regulates goblet cell mucus secretion in mice, and the colon mucus layer is thinner in *Bcl2*^AAA^ mice than wild-type mice (30). Secretory autophagy in Paneth cells also promotes resistance to *S.* Typhimurium in mice (39). Although the role of BCL2 in secretory autophagy is unclear, it remains possible that defective secretory autophagy contributes to the dissemination phenotypes in the *Bcl2*^AAA^ mice. Finally, autophagy controls antigen presentation and cytokine production in nonepithelial immune cells (40, 41), which could also contribute to the reduced resistance to infection in *Bcl2*^AAA^ mice.

The central role of the BCL2–BECN1 complex in enterocyte antibacterial autophagy could present opportunities for pharmacologic targeting of autophagy to enhance antibacterial immunity. Autophagy is amenable to pharmacological targeting in other disease settings, including viral infections, cancer, and neurodegenerative disease (42, 43). For example, a BECN1 peptide induces antiviral autophagy that inhibits the replication of several viruses (44). We speculate that targeted disruption of the BCL2–BECN1 complex could also enhance enterocyte autophagy to promote clearance of invading bacteria during an intestinal infection.

## Materials and Methods

### Mice.

Wild-type C57BL/6, *Myd88^−/−^* (45), *Myd88^fl/fl^* (46), *Myd88*^ΔIEC^ (29), *Bcl2*^AAA^ (13), *Becn1*^F121A^ (26, 27), and *Jnk1^−/−^* (47) mice were maintained in the barrier at the University of Texas Southwestern Medical Center. *Myd88*^ΔIEC^ mice were generated by crossing *Myd88^fl/fl^* mice (46), with a mouse expressing Cre recombinase under the control of the intestinal epithelial cell-specific Villin promoter (Villin-Cre mice; Jackson Laboratory) (48) as previously described (29). Germ-free C57BL/6 and *MyD88^−/−^* mice were maintained in isolators at the University of Texas Southwestern Medical Center. *Jnk1^−/−^* mice (B6.129S1-Mapk8^tm1Flv/J^) were obtained from The Jackson Laboratory. *Bcl2*^AAA^ and *Becn1*^F121A^ mice were gifts from Beth Levine at the University of Texas Southwestern Medical Center. For all experiments, 6- to 8-wk-old mice were used and littermate controls were examined. All experiments were performed using protocols approved by the Institutional Animal Care and Use Committees of the University of Texas Southwestern Medical Center.

### Bacterial Strains.

*S.* (SL1344) (49) and *S.* Typhimurium–GFP (a gift from V. Sperandio, University of Wisconsin, Madison) (5) were grown in Luria broth at 37 °C with antibiotics (50 μg/mL streptomycin for *S.* Typhimurium SL1344 and 100 μg/mL ampicillin, 50 μg/mL streptomycin for *S.* Typhimurium–GFP).

### Antibodies and Reagents.

Rabbit anti-LC3 antibodies (1:300 for immunostaining and 1:1,000 for immunoblot) were from Novus Biologicals (NB100-2220 used for immunostaining and NB 100-2331 used for immunoblot). Goat anti-GFP (1:1,000 for immunostaining), donkey anti-goat immunoglobulin G (IgG) (conjugated to DyLight 488, 1:500) and Cy3 donkey anti-rabbit IgG (1:500) were from Abcam.

For immunoprecipitations, mouse anti-BECN1 (E-8) (1:500; Santa Cruz) was used for BECN1 immunoprecipitations in Figs. 1*H* and 5*K*; rabbit anti-BECN1 (1:500; Novus Biologicals; cat. no. NB500-249) was used for immunoblot detection of BECN1 in immunoprecipitations shown in Fig. 1*H*; and rabbit anti-BECN1 (H-300) (1:500; Santa Cruz; cat. no. SC-11427) was used for immunoblot detection in immunoprecipitations shown in Fig. 5*K*. Goat anti-rabbit IgG-HRP (1:500; Abcam; cat. no. AB6721) was used to detect both the rabbit anti-BECN1 antibodies. Other immunoblot antibodies were mouse anti-BCL2 HRP (1:100; Santa Cruz), rabbit anti-p-BCL2 (Ser70) (1:200; Santa Cruz), mouse anti-JNK1 (1:200; Santa Cruz), anti-p-JNK (1:200; Santa Cruz), and mouse anti-Mito70 (1:10,000; Abcam).

### Organoid Culture.

Organoids were cultured from small intestinal crypts recovered from 6- to 8-wk-old mice, using a protocol based on the methods described in ref. 23. Small intestines were rinsed with phosphate buffered saline (PBS), fat was removed, and the intestines were cut longitudinally. The intestines were then cut into 5 to 10 cm pieces and washed in PBS. Villi were scraped using a glass microscope slide by firmly drawing the edge of the slide along the entire length of the intestine. The intestines were then cut into 1 cm pieces which were placed in 20 mL of cold PBS and shaken vigorously. The pieces were transferred to a new tube with PBS and the shaking was repeated until the supernatant appeared clear. The intestinal pieces were transferred to a new tube containing 20 mL of 2 mM ethylenediaminetetraacetic acid (EDTA) in PBS and rotated at 4 °C for 30 min. After vigorously shaking, the pieces were transferred to a new tube containing 20 mL of 5 mM EDTA in PBS and rotated at 4 °C for 15 min. Crypts were eluted by vigorous shaking and elution was confirmed by microscopy. Supernatants were combined and centrifuged at 300 g for 5 min. The pellets were resuspended in medium (Dulbecco’s Modified Eagle Medium (DMEM)+GlutaMAX™ [Gibco] with 10% fetal bovine serum) and strained through a 70 μm filter. The centrifugation steps were repeated two to three times until the supernatant was clear. The number of crypts was calculated by mixing with trypan blue and counting under a light microscope. The pellets were resuspended in 50 μL IntestiCult™ Organoid Growth Medium (mouse; Stem Cell Technologies) and mixed with 200 μL MatriGel (BDSciences). The mixture was transferred into six wells of a prewarmed 24-well plate and routinely yielded 100 to 500 organoids per well. The plate was incubated at 37 °C for 10 to 20 min until the MatriGel solidified. Then, 500 μL of IntestiCult™ Organoid Growth Medium was added to each well and incubated at 37 °C under 5% CO_2_. The organoid growth medium was changed every 2 to 3 d.

### *S.* Typhimurium Infections of Mice and Small Intestinal Organoids.

Conventional mice were treated with antibiotics (2 mg/mL ampicillin, 2 mg/mL streptomycin; 200 μL per mouse delivered intragastrically). The next day mice were infected intragastrically by gavage with 5 × 10^9^ CFU of *S.* Typhimurium (SL1344) or *S.* Typhimurium–GFP per mouse. Bacterial loads in the small intestine, colon, liver, spleen, and MLNs were determined by dilution plating after a 24-h infection by plating on Luria broth plates containing antibiotics (50 μg/mL streptomycin for *S.* Typhimurium SL1344 and 100 μg/mL ampicillin, 50 μg/mL streptomycin for *S.* Typhimurium–GFP).

For organoids, after 7 to 10 d of culture in MatriGel with the culture medium (IntestiCult™ Organoid Growth Medium, StemCell Technologies), 500 μL of cold medium (DMEM + 10% fetal bovine serum) was added to each well, and the mixture was transferred to Eppendorf tubes after the MatriGel was melted. Organoids were collected by centrifugation and disrupted by pipetting using bent 200 μL tips. Disrupted organoids were washed three times in DMEM medium and returned to each well along with 500 μL IntestiCult™ Organoid Growth Medium (StemCell Technologies). Then, 1 × 10^6^ CFU of *S.* Typhimurium–GFP was added to each well for infection. Gentamicin (200 μg/mL) was added after 1 h of infection. Organoids were infected for another 3 h and collected for analysis.

Following infection, organoids were thoroughly washed using 3% fetal bovine serum in PBS to remove extracellular bacteria. Organoids were weighed in Eppendorf tubes; samples weighed ~20 to 30 mg. Then, 100 μL of T-PER buffer (Thermo Scientific) was added to each tube and lysed for 10 min on ice. Samples were diluted 1:10 and plated on Luria broth plates containing antibiotics (100 μg/mL ampicillin, 50 μg/mL streptomycin). *S.* Typhimurium–GFP colonies on each plate were counted the next day.

### Immunofluorescence Microscopy of Mouse Small Intestines and Organoids.

Small intestines were treated with Bouin’s fixative overnight at 4 °C and embedded in paraffin. After sectioning, the tissues were washed twice for 10 min in xylene, twice for 3 min in 100% ethanol, twice for 3 min in 95% ethanol, twice for 3 min in 70% ethanol, and then rinsed in deionized water for 5 min. Sections were boiled in 10 mM sodium citrate (pH 6) for 25 min, washed in PBS, and blocked for 15 min in 2% bovine serum albumin (BSA)/PBS, followed by 15 min in 1% BSA, 0.3% Triton X-100 in PBS. Organoids were fixed in 4% formaldehyde and embedded in clear frozen section compound (VWR). After sectioning, the tissues were washed in PBS and blocked with 2% BSA/PBS for 15 min and 1% BSA/0.3%Tween-20/PBS for 15 min. Sections were incubated with primary antibody overnight. Rabbit anti-LC3 antibodies were used to visualize autophagosomes and goat anti-GFP antibodies were used to visualize GFP-labeled *Salmonella* bacteria. After washing in Tris-buffered saline and Tween-20 (TBS-T: 50 mM Tris pH 7.6, 150 mM NaCl, and 0.05% Tween-20), sections were incubated with Cy3 donkey anti-rabbit IgG (Invitrogen) and DyLight 488-conjugated anti-goat IgG (Invitrogen) for 1 h at 25 °C and counterstained with DAPI. Images were captured on a Zeiss AxioImager M1 Microscope.

### Immunoblotting.

Ileums were flushed with ice-cold PBS, and epithelial cells were lysed in situ for 5 min with Tissue Protein Extraction Reagent (T-PER, Thermo Scientific) supplemented with a Complete Protease Inhibitor Cocktail (Roche) and Halt™ Phosphatase Inhibitor Cocktail (Thermo Fisher Scientific). For organoids, 500 μL cold DMEM medium (with 10% fetal bovine serum) was added to each well, and organoids were transferred to Eppendorf tubes after the MatriGel was melted. Organoids were washed three times with cold PBS buffer, 100 μL T-PER buffer was added to each tube, and the organoids were lysed for 5 min. Lysates were centrifuged at 4 °C for 5 min, added sodium dodecyl sulfate (SDS) loading buffer and boiled, and analyzed by immunoblotting.

For immunoblotting, 75 µg of total protein was loaded onto 4 to 20% gradient polyacrylamide gel (BioRad) and transferred to a polyvinylidene fluoride (PVDF) membrane. Membranes were blocked with blotting-grade blocking buffer (BioRad) in TBS with 0.1% Tween-20. Membranes were incubated at room temperature for 1 h and overnight at 4 °C with primary antibody. After washing with TBS with 0.1% Tween-20, membranes were incubated with secondary antibody. Membranes were visualized using a Bio-Rad ChemiDoc™ Touch system, and band density was quantified using Bio-Rad Image Lab Software 5.2.1.

### Coimmunoprecipitation.

BECN1/BCL2 coimmunoprecipitation assays were performed using small intestine epithelial cell lysates which were prepared as described above under Immunoblotting. The protein concentrations of the cell lysates were measured using the Pierce™ BCA Protein Assay Kit (Thermo Scientific) and adjusted to 5 mg/mL. A volume of 5 μL of mouse anti-BECN1 (Santa Cruz) antibody was added to the cell lysate and incubated at 4 °C for 1 h. The cell lysate/antibody mixture was then added to 20 μL Pierce™ Protein A Agarose beads (Thermo Scientific) and incubated at 4 °C overnight. The next day, the beads were washed three times with T-PER buffer (Thermo Scientific). Then, 50 μL of SDS loading buffer was added to the beads and boiled for 5 min prior to immunoblotting analysis. Mouse anti-BCL2-HRP (1:100; Santa Cruz) and rabbit anti-BECN1 (1:500; Novus Biologicals; Fig. 1*H*) or rabbit anti-BECN1 (1:500, Santa Cruz; Fig. 5*K*) and were used for immunoblots. The rabbit anti-BECN1 antibodies were detected with HRP-conjugated goat anti-rabbit IgG (Abcam).

### TUNEL Assay.

The terminal deoxynucleotidyl transferase deoxyuridine triphosphate (dUTP) nick end labeling (TUNEL) reaction was performed to compare the apoptosis status of the intestinal epithelial cells of wild-type and *Bcl2^AAA^* mice. Fragments of ileum were treated with Bouin’s fixative overnight at 4 °C and embedded in paraffin. After sectioning, the tissue sections were dewaxed and rehydrated by incubating twice for 10 min in xylene, twice for 3 min in 100% ethanol, twice in 95% ethanol, twice in 70% ethanol, and then rinsed in deionized water for 5 min. Sections were boiled in 10 mM sodium citrate (pH 6) and washed three times in PBS. Excess buffer was carefully blotted away and 50 μL of proteinase K solution (20 μg/mL in 10 mM Tris-HCI, pH 7.4) was added to cover the sections. The sections were incubated for 15 min at room temperature, and the slides were washed 3 × 5 min with PBS. Two slides were set aside for positive controls and incubated with DNase I solution (2,500 U/mL, Thermo Fisher) for 10 min at room temperature to induce DNA strand breaks. The slides were rinsed twice with PBS, and the area around the samples was dried. The In Situ Cell Death Detection Kit, Fluorescein (Roche), was used for apoptosis detection. For two negative controls, 100 μL Label Solution (vial 2) was removed. Then, 50 μL Enzyme Solution (vial 1) was added to the remaining 450 μL Label Solution (vial 2) to obtain 500 μL of TUNEL reaction mixture. Fifty microliters of the TUNEL reaction mixture was added to each sample and covered with a coverslip. Slides were incubated in a humidified chamber for 1 h at 37 °C in the dark. The slides were washed with PBS, air dried, and counterstained with DAPI (DAPI Fluoromount-G, SouthernBiotech). Tissue sections were covered with coverslips and observed under a fluorescence microscope (Zeiss AxioImager M1).

### Intestinal Permeability Assay.

Overall intestinal permeability in wild-type and *Bcl2*^AAA^ mice was measured by oral administration of fluorescein isothiocyanate (FITC)-dextran. FITC-dextran (4 kDa; 80 mg/mL in PBS; Sigma-Aldrich) was orally administered to the mice (600 mg/kg body weight). Mice were killed 4 h later and sera were collected. The sera were centrifuged for 20 min at 3,000 rpm at 4 °C, and the supernatants were collected. Serum FITC-dextran levels were determined by fluorescence microplate assay against a FITC-dextran standard curve.

### Quantification and Statistical Analysis.

Statistical details of experiments can be found in the figure legends, including how significance was defined and the statistical methods used. Data represent mean ± SEM. No method was used to predetermine sample size. Blinding was not performed for these experiments. Formal randomization techniques were not used; however, mice were allocated to experiments randomly and samples were processed in an arbitrary order. All statistical analyses were performed with GraphPad Prism software. To assess the statistical significance of a difference between two groups of mice, we used two-tailed Student’s *t* tests. The only mice excluded from experiments were mice that died during the experiment.

## Supplementary Material

Appendix 01 (PDF)

## Data Availability

All study data are included in the article and/or *SI Appendix*.
