# Supplementary material for: BCL2 regulates antibacterial autophagy in the intestinal epithelium
Source: Proc Natl Acad Sci U S A. 2024 Nov 27;121(49):e2410205121. doi: 10.1073/pnas.2410205121 (PMC11626146; doi:10.1073/pnas.2410205121)
Supplement: Supplementary file 1 — Appendix 01 (PDF) [file pnas.2410205121.sapp.pdf]

PNAS

Supporting Information for

BCL2 regulates antibacterial autophagy in the intestinal epithelium

Yun Li, Shai Bel, Jamaal L. Benjamin, Kelly A. Ruhn, Brian Hassell, Cassie L. Behrendt, Zheng Kuang, and Lora V. Hooper

Corresponding authors: Yun Li and Lora V. Hooper

Email: yun2.li@utsouthwestern.edu; lora.hooper@utsouthwestern.edu

This PDF file includes:

Figures S1 to S5

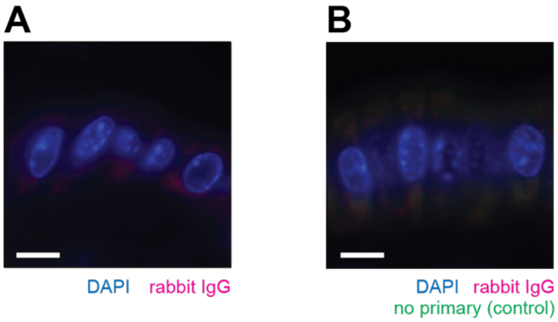

Figure S1. Immunofluorescence controls for Figures 1 through 5.

(A) Immunofluorescence detection of uninfected mouse small intestinal villus tips with control rabbit IgG and Cy3-conjugated donkey anti-rabbit IgG secondary antibody. Scale bar, 10 μm.

(B) Immunofluorescence detection of *S*. Typhimurium-GFP-infected mouse small intestinal villus tips with control rabbit IgG and Cy3-conjugated donkey anti-rabbit IgG secondary antibody. The tissue was also detected with DyLight 488-conjugated donkey anti-goat IgG secondary antibody as a “no primary antibody” control for detection of *S*. Typhimurium-GFP. Scale bar, 10 μm. Nuclei are stained with DAPI.

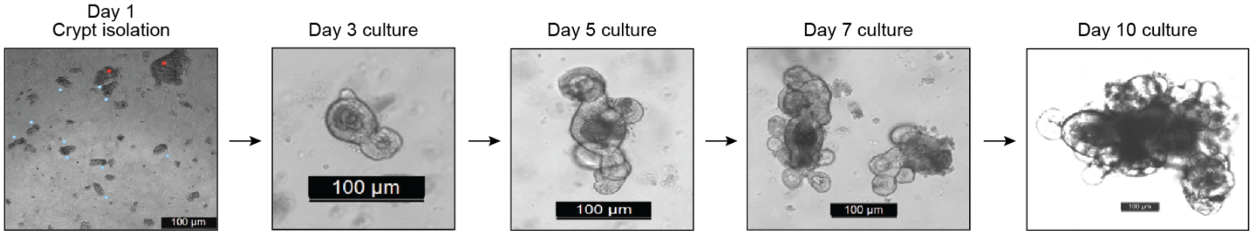

Figure S2. Organoid culture from mouse small intestine.
Organoids were cultured from crypts recovered from mouse small intestine as described in Materials and Methods. Day 1 shows recovered crypts. Blue dots indicate crypts with the potential to develop into organoids. Red dots indicate clumps of epithelial cells. 3-day, 5-day, 7-day and 10-day organoid cultures are shown. Scale bars, 100 μm.

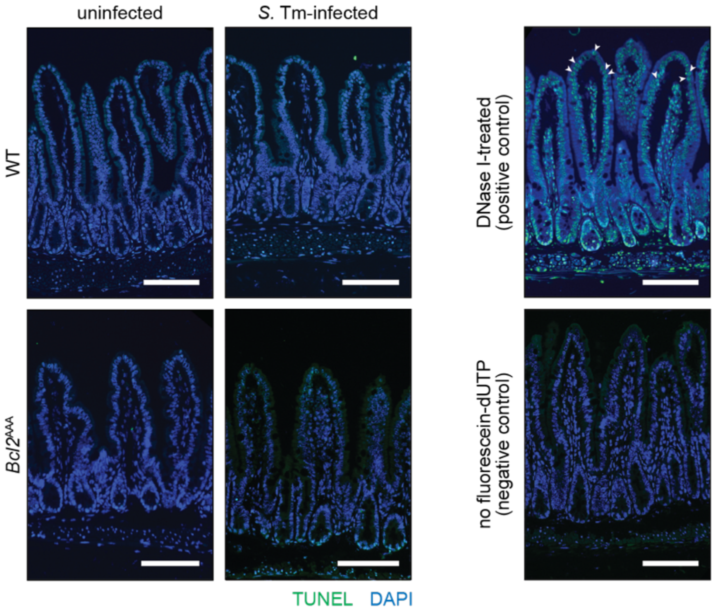

Figure S3. The BCL2^{AAA} mutant protein retains its anti-apoptotic function in intestinal epithelial cells.
BCL2 has dual functions by opposing both autophagy and apoptosis (12). To test whether the *Bcl2*^{AAA} mutation affects its anti-apoptotic function in the intestine, we performed the terminal deoxynucleotidyl transferase dUTP nick end labeling (TUNEL) reaction to detect apoptotic cells in the small intestines of wild-type (WT) and *Bcl2*^{AAA} mice. Mice were either left uninfected or were infected with *Salmonella* Typhimurium (SL1344) (5×10^9 CFU per mouse for 24 hours). The TUNEL reaction was conducted with fluorescein-dUTP. DNase I treatment introduced double-stranded DNA breaks into the sample tissue and was used as a positive control. Arrowheads show examples of TUNEL$^+$ cells in the positive control. The labeling solution with the fluorescein-dUTP omitted was used as a negative control. Scale bars, 100 μm. Images are representative of images from n=5 mice.

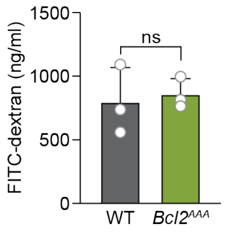

Figure S4. ***Bcl2*^{AAA} mice do not show altered intestinal permeability.**
To measure intestinal permeability, FITC-dextran (600 mg/kg body weight; 4 kDa; Sigma) was delivered orally to wild-type (WT) and *Bcl2*^{AAA} mice, and serum FITC-dextran levels were determined by fluorescence microplate assay against a standard curve. Each data point represents one mouse (n=3 mice per group). ns, not significant by Student's *t* test. Results are representative of three independent experiments.

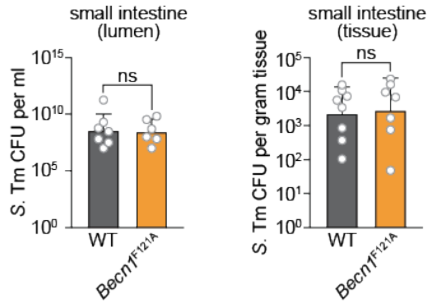

Figure S5. *Becn1*$^{\mathbf{F121A}}$ mice do not show altered numbers of small intestinal *Salmonella* Typhimurium.
Wild-type (WT) and *Becn1*^{F121A} littermates were intragastrically infected with 5×10^9 CFU of *S*. Typhimurium for 24 hours or were left uninfected. Bacterial counts in the small intestinal lumen or tissue were determined by dilution plating. Each point represents an individual mouse, and data are from three independent experiments. Geometric means ± SEM are plotted; ns, not significant by Student's *t* test.
